# LC-MS/MS and LC-UV Determination of Moniliformin by Adding Lanthanide Ions to the Mobile Phase

**DOI:** 10.3390/toxins11100570

**Published:** 2019-09-29

**Authors:** Terenzio Bertuzzi, Silvia Rastelli, Annalisa Mulazzi, Amedeo Pietri

**Affiliations:** Department of Animal Science, Food and Nutrition-DIANA, Faculty of Agriculture, Food and Environmental Sciences, Università Cattolica del Sacro Cuore, Via Emilia Parmense, 84-29122 Piacenza, Italy; silvia.rastelli@unicatt.it (S.R.); annalisa.mulazzi@unicatt.it (A.M.); amedeo.pietri@unicatt.it (A.P.)

**Keywords:** moniliformin, lanthanide complexes, LC-MS/MS, LC-UV

## Abstract

An innovative chromatographic analysis was developed for the determination of moniliformin (MON). Because of its ionic nature, MON is weakly retained in reversed-phase chromatography and the separation may be tricky. Nevertheless, this technique is normally used either with the formation of ion pairs or employing specific RP columns for polar compounds, or combining anion exchange and hydrophobic interactions. Hydrophilic interaction chromatography (HILIC) was also used, but a non-negligible peak tailing was observed. Besides its ionic nature, MON is a di-ketone and di-ketones, mainly β-di-ketones, can easily form complexes with lanthanide ions. Then, in this work the addition of lanthanide ions to the mobile phase was investigated, aiming at improving peak shape and MON separation. La^3+^, Tb^3+^ or Eu^3+^ aqueous solutions were used as mobile phase and MON was chromatographed using a LC-NH_2_ column. The probable formation of coordination complexes lanthanide-MON in the HPLC mobile phase allowed to obtain a symmetrical peak shape and a satisfactory chromatographic separation by both mass spectrometry (MS/MS) and UV detection. Finally, a suitable extraction and purification method for MON determination in cereal samples was developed.

## 1. Introduction

**Figure 1:**
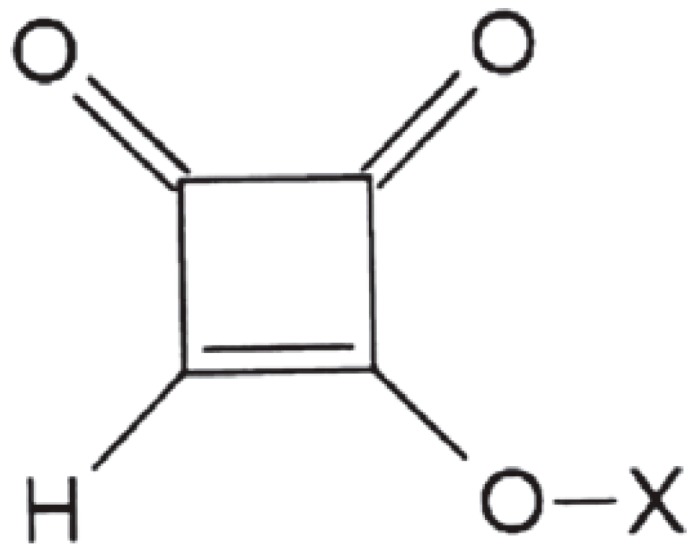
Chemical structure of moniliformin (X = H, Na or K).

**Figure 2:**
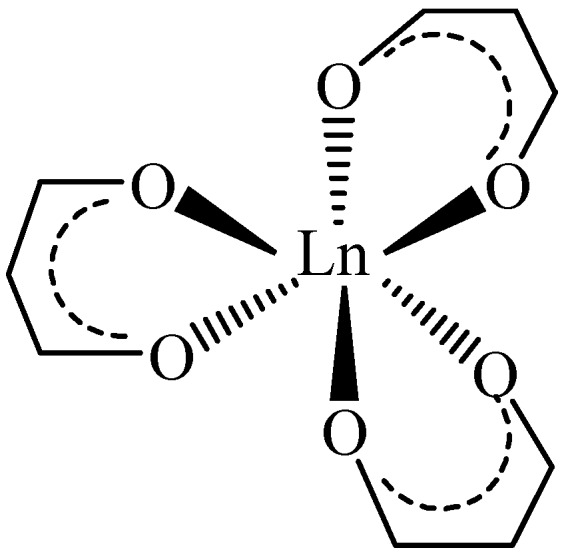
Structure of a [Ln-(β-di-ketone)_3_] complex (Ln: lanthanide ion).

Moniliformin (MON) is a *Fusarium* mycotoxin often occurring in cereals; it is mainly produced by *F. avenaceum, proliferatum, subglutinans, tricinctum* and *verticilloides* [1]. MON is a small, highly polar, acidic molecule and because of the low pKa value (<1.7) of the free acid, MON occurs as a water-soluble sodium or potassium salt [2]. MON is toxic to experimental animals, causing myocardial degeneration, muscular weakness and respiratory distress [3,4]; the European Food Safety Authority (EFSA) Panel on Contaminants in the Food Chain (CONTAM) indicated haematotoxicity and cardiotoxicity as major adverse health effects of MON [5]. At present, no specific maximum levels for MON in food and feed have been set by EU legislation. MON was detected worldwide in several cereal crops at different concentration levels with values up to 2606 µg kg^−1^ in maize and 326 µg kg^−1^ in wheat produced, respectively, in Italy and in the Netherlands being reported [1,6,7,8,9,10,11]. However, EFSA recommended the collection of more occurrence data on MON in foods and feeds to enable a comprehensive risk assessment for humans, and for farm and companion animals [5]. The same report recommended the development of validated analytical methods for MON determination. Because of its ionic nature, MON is weakly retained by reversed-phase (RP) chromatography. Nevertheless, this technique is normally used either with the formation of ion pairs [12] or employing specific RP columns for polar compounds, or combining anion exchange and hydrophobic interactions [9,13,14]. Hydrophilic interaction chromatography was also used, but a non-negligible peak tailing was observed [8]. In this work, a new approach was evaluated; besides its ionic nature, MON is a di-ketone (1-hydroxycyclobut-1-ene-3,4-dione. As seen in Figure 1, di-ketones, mainly β-di-ketones, can easily form complexes with lanthanide ions (as Lanthanum La^3+^, Terbium Tb^3+^ or Europium Eu^3+^), linking the metallic ion through the oxygen atoms [15]. Generally, three di-ketones are linked to one lanthanide ion (Figure 2).

Then, in this work the addition of lanthanide ions to the mobile phase was investigated, aiming at improving peak shape and MON separation. MON determination was carried out using both UV and mass spectrometry (MS/MS) detection. UV detection was used for its major flexibility during the development of the method and MS/MS detection for its major accuracy and lower detection limits. Finally, a suitable extraction and purification method for MON determination in cereal samples was developed.

## 2. Results and Discussion

### 2.1. Development of the Chromatographic Method

**Figure 3:**
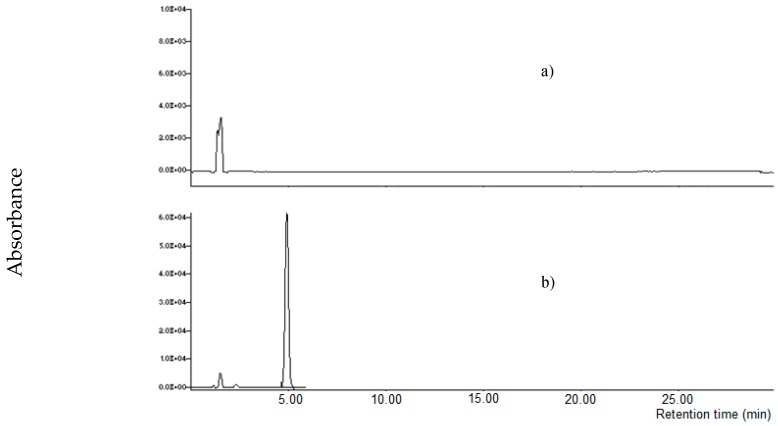
Chromatographic separation of moniliformin (MON) standard (1000 µg l^−1^) on a LC-NH_2_ column using water (**a**) or 10 mM La^3+^ aqueous solution (**b**) in the mobile phase.

It is known that MON is weakly retained in RP chromatography, recently, specific columns for polar compounds were used for its determination. Initially, four columns were tested using an HPLC-UV instrument: an RP-8 (Lichrospher, 5 μm particle size, 125 × 4 mm i.d., Merck, Darmstadt, Germany), a X-Select HSS T3 (RP-18 with low ligand density, 2.5 µm particle size, 100 × 2.1 mm i.d., Waters Corporation, Milford, MA, USA), a XBridge BEH Amide column (2.5 µm particle size, 100 × 2.1 mm i.d., Waters Corporation, Milford, MA, USA) and a Supelcosil LC-NH_2_ column (250 × 3 mm, 5 µm, Supelco, Bellefonte, PA, USA); a mixture acetonitrile:water 20 + 80 *v/v* at a flow rate of 1.0 mL min^-1^ was used as mobile phase. A MON standard at 1000 µg L^−1^ was injected; MON was weakly retained by RP-8, X-Select HSS T3 and BEH Amide columns (retention time lower than 2.0 min); on the other hand, MON was strongly retained by the LC-NH_2_ column (no peak was observed until 30 min). Successively, the mobile phase was substituted with acetonitrile: 10 mM LaCl_3_·7H_2_O 20 + 80 *v/v*; no relevant difference of the retention time was observed for RP-8, X-Select HSS T3 and BEH Amide column, while MON was eluted by the LC-NH_2_ column at 4.9 min (Figure 3).

**Figure 4:**
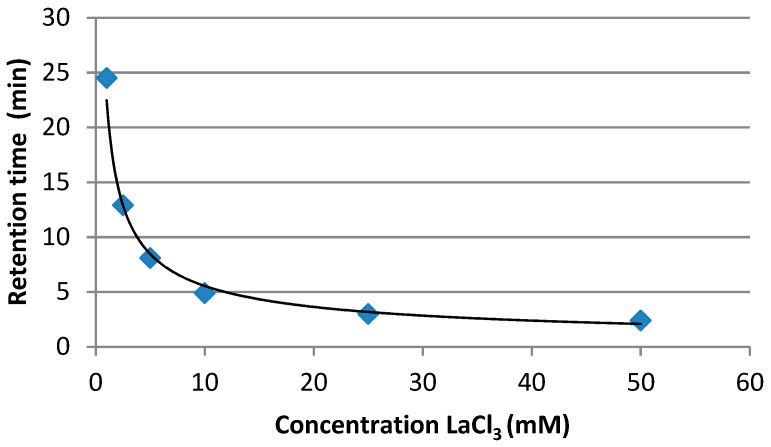
Variation of retention time depending on concentration of LaCl_3_ (mM) in the mobile phase.

Five calibration standards were injected (20, 100, 250, 500 and 1000 µg L^−1^), showing a satisfactory calibration curve (*R*^2^ = 0.998). Successively, increasing concentrations from 1 to 50 mM of LaCl_3_·7H_2_O in the mobile phase were tested and a decrease of the retention time was observed (Figure 4).

**Figure 5:**
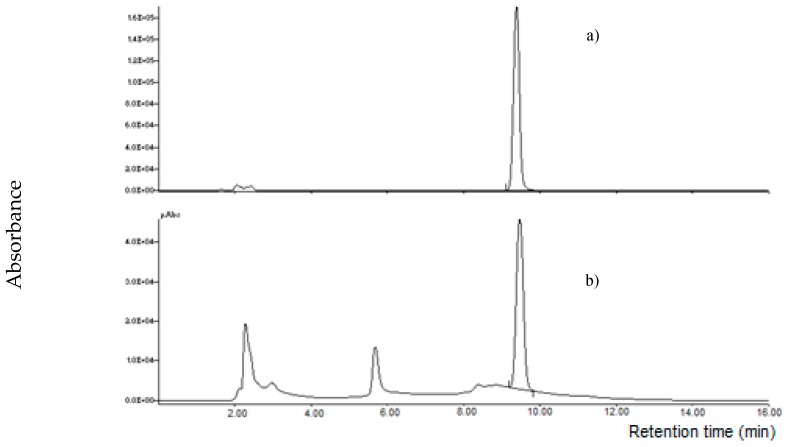
Chromatograms of: (**a**) MON standard (1000 µg L^−1^); (**b**) maize spiked extract (250 µg L^−1^). Chromatographic separation was carried out using linear gradient acetonitrile: 2.5 mM LaCl_3_·7H_2_O solution; detection at 260 nm (UV).

Finally, a satisfactory chromatographic separation was obtained applying a linear gradient acetonitrile:2.5 mM LaCl_3_·7H_2_O (Figure 5); a low LaCl_3_·7H_2_O concentration was preferred in order to delay MON elution and avoid co-elution with other substances.

**Figure 6:**
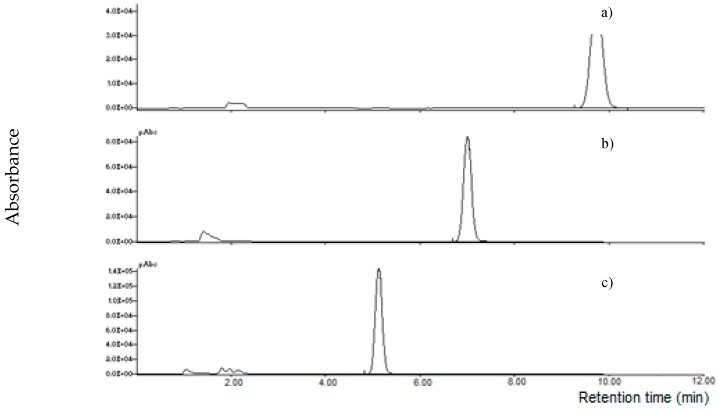
Chromatographic separation of MON standard (1000 µg L^−1^) on LC-NH_2_ column using: (**a**) 2.5 mM Tb^3+^ aqueous solution:acetonitrile 4 + 6 *v/v*; (**b**) 2.5 mM Tb^3+^ aqueous solution:acetonitrile 6 + 4 *v/v*; (**c**) 2.5 mM Tb^3+^ aqueous solution:acetonitrile 8 + 2 *v/v*, as mobile phase.

Similar results were obtained using Tb^3+^ or Eu^3+^; MON was detected at a retention time of 7.0 min using acetonitrile:2.5 mM TbCl_3_·6H_2_O 60 + 40 *v/v* (Figure 6). Because of its easy availability and lower cost, it was preferred to use LaCl_3_·7H_2_O for the following quantitative MON analyses.

The chromatograms indicate a coordination of MON with lanthanide metals during the separation, resulting in a stronger affinity of MON for the mobile phase and a consequent faster elution from the LC-NH_2_ column. Furthermore, increasing the concentration of La^3+^, a higher MON affinity for the mobile phase is favoured and therefore shorter retention times are shown. It is known that lanthanide ions can form coordination complexes with di-ketones [15], as well as with other organic compounds. Recently, a coordination between cyclopiazonic acid, a neurotoxin and lanthanide metals was reported by Maragos [16].

**Figure 7:**
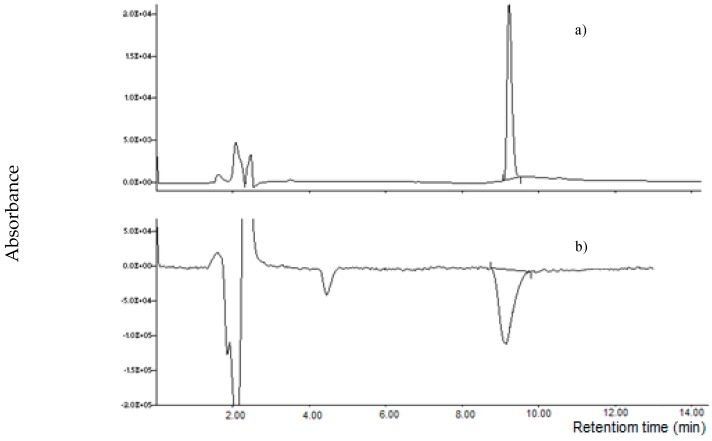
Chromatograms of MON standard (1000 µg L^−1^) detected by UV at 260 nm (**a**) and fluorimeter detector at λ_ex_ = 260 nm; λ _em_ = 550 nm (**b**).

Generally, the luminescence of some lanthanides, such as Terbium and Europium, can be enhanced by interaction with selected molecules. For example, Terbium was used in post-column HPLC separation to increase the luminescence of ochratoxin A [17]. The luminescence is greatly influenced by the solvent and in particular by the presence of water, as well as by the lanthanide concentration and the chelate/lanthanide ratio [16]. Using a mobile phase 2.5 mM Tb^3+^ aqueous solution:acetonitrile 4 + 6 *v/v*, we replaced the UV with a fluorimetric detector (λ_ex_ = 260 nm; λ_em_ = 550 nm) in order to evaluate a possible fluorescence. However, the results showed a negative peak at the retention time of MON detected by the fluorimeter, showing a decrease of fluorescence of the baseline (Figure 7). During the chromatographic separation, water and lanthanide concentrations are very much higher than MON concentration. These conditions do not seem to favour an increase of fluorescence, as already reported [16,18,19].

**Figure 8:**
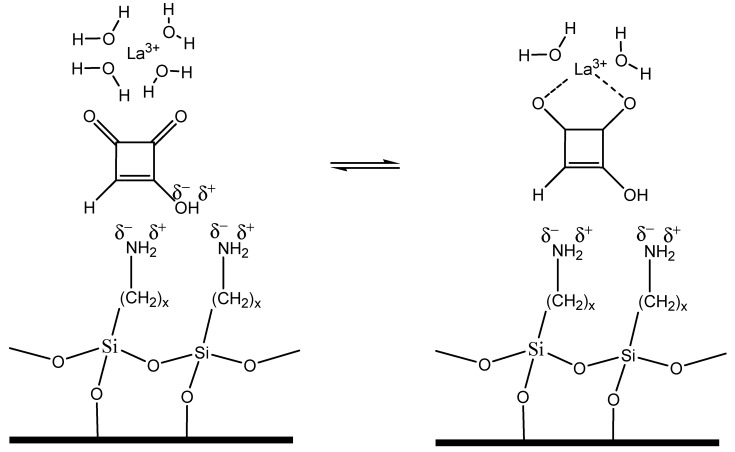
Possible structure of the coordination complex during chromatographic separation in presence of La^3+^ in the mobile phase.

The process of MON separation adding lanthanide ions in the mobile phase can be speculated considering the theory of ligand exchange chromatography (LEC), a chromatographic process in which complex-forming compounds are separated through the formation and breaking of labile coordinate bonds to a central metal atom, coupled with partition between a mobile and a stationary phase [20]. Based on LEC theory, the coordination of lanthanide ions with water in the mobile phase, can be partially replaced with the coordination to MON, resulting in the formation of mixed coordination complexes (Figure 8). These coordination complexes are kinetically weak, their formation and dissociation is fast and can be described by the following reversible reaction:

MON + [Ln(H_2_O)*_n_*] ↔ [Ln(MON)(H_2_O)*_n_*_−1_] + H_2_O Ln—lanthanide

This process could explain the higher MON affinity for the mobile phase and the faster elution, when lanthanide concentration is increased. In absence of lanthanide ions in the mobile phase, MON is strongly retained by the stationary phase of the LC-NH_2_ column.

**Figure 9:**
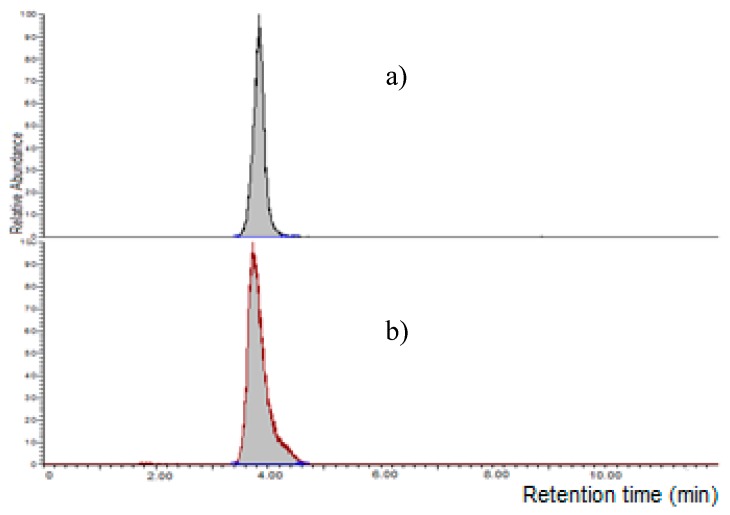
Chromatograms of: (**a**) MON standard (100 µg L^−1^); (**b**) naturally contaminated maize sample (1637 µg kg^−1^). Chromatographic separation was carried out using a linear gradient methanol: 25 mM ammonium acetate containing 1.25 mM LaCl_3_·7H_2_O; detection by MS/MS (MRM transition *m/z* 97 >41).

Finally, the chromatographic analysis was carried out using a mass spectrometric detector (MS/MS, triple quadrupole, Thermo Fisher Scientific, San Jose, CA, USA) in order to obtain high accuracy and lower detection limits; a very low LaCl_3_·7H_2_O concentration was used (1.25 mM) to avoid troubles during the ionisation. MON was chromatographed on a 75 × 3 mm, 3 µm LC-NH_2_ column (Supelco) and detected at 4.4 min, improving the limit of detection (Figure 9).

### 2.2. Development of an Extraction and Purification Method for Cereal Samples

Generally, MON was extracted using a mixture acetonitrile:water 84:16 (as for tricothecenes). Considering that MON is highly water-soluble, Barthel et al. [10] and Herrera et al. [9] recently increased the percentage of water in the extraction mixture, using acetonitrile:water 50 + 50 *v/v* or pure water, obtaining higher extraction yields. Since in our tests the extract using water 100% was turbid, probably for the presence of other large polar molecules, the mixture acetonitrile:water 50 + 50 *v/v* was preferred. As regards the purification step, several authors used either a SAX-like SPE [9] or a MycoSep^®^ MON 240 column [8,13]. However, Herrera et al. [10] did not obtain satisfactory recoveries using these columns and consequently no clean-up step was carried out in their method. In this work, we previously tested MycoSep^®^ MON 240 (Romer Labs, Getzersdorf, Austria), MAX, WAX and HLB OASIS columns (Waters Corporation, Milford, MA, USA) and the Quechers procedure; in all tests, unsatisfactory purification or low recoveries were obtained. Moreover, we confirmed that the evaporation under N_2_ flow decreased the recovery, as reported by Herrera et al. [10]. Finally, a LC-NH_2_ column (Supelco) was tested, in order to retain MON and elute it using a lanthanide ion solution, as for the HPLC separation. This clean-up step was only introduced for HPLC-UV determination, for LC-MS/MS analysis, the presence of high concentration of La^3+^ (12.5 mM) in the purified extract worsened MON detection.

### 2.3. Performances of the Method

The result of the considered parameters are shown in Table 1. Despite the omission of clean-up step for LC-MS/MS analysis, the matrix effect, calculated at the concentration of 100 µg L^−1^ (800 µg kg^−1^ for a cereal sample), was low, close to 4% and 6% for wheat and maize samples, respectively.

A satisfactory linearity was calculated for both LC-MS/MS (*R*^2^ = 0.997) and HPLC-UV (*R*^2^ = 0.998).

The limit of detection (LOD), for a cereal sample, was 10 and 80 µg kg^−1^ using LC-MS/MS and HPLC-UV, respectively. The decision limit (CCα) and the detection capability (CCβ) were 10 and 18 µg kg^−1^ for LC-MS/MS, 80 and 136 µg kg^−1^ for LC-UV.

Skipping the clean-up step (LC-MS/MS analysis), the global average recoveries were 97.1% ± 4.3% and 96.4% ± 5.1% for wheat and maize, respectively. Using purification step (UV analysis), the average recoveries were: 86.4% ± 6.4% and 89.8% ± 4.5% for wheat (at 100 and 500 µg kg^−1^, respectively), 83.6% ± 5.9% and 86.1% ± 5.1% for maize (at 250 and 1000 µg kg^−1^, respectively).

Finally, the standard deviation obtained by repeatability tests was less than 9.2%.

### 2.4. MON Occurrence in Cereal Samples

**Figure 10:**
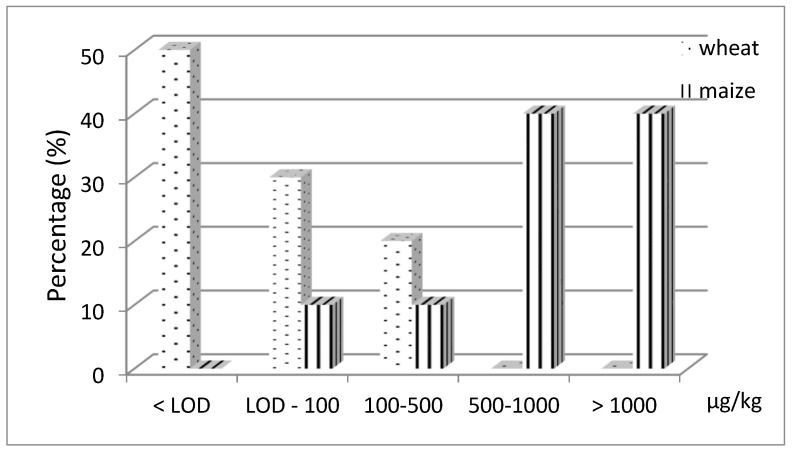
Frequency of occurrence (%) of MON in wheat (*n* = 10) and maize (*n* = 10) collected in northern Italy.

MON occurred in 100% and 50% of maize and wheat samples, respectively. The levels of contamination, corrected for the recovery percentage, ranged between 38 and 3629 µg kg^−1^ (the last value was obtained after dilution of the sample extract) for maize (in 4 samples MON exceeded 1000 µg kg^−1^), <10 and 481 µg kg^−1^ for wheat samples (Figure 10).

## 3. Materials and Methods

### 3.1. Reagents and Standards

Chemicals and solvents used for extraction and clean-up were ACS grade or equivalent (Carlo Erba, Milan, Italy); deionised water was purified through a Milli-Q treatment system (Millipore, Bedford, MA, USA). For HPLC and LC-MS/MS analysis, water, methanol and acetonitrile were HPLC grade (Merck, Darmstadt, Germany). MON (as sodium salt), Lanthanum (III) chloride heptahydrate (LaCl_3_·7H_2_O), Terbium (III) chloride hexahydrate (TbCl_3·_6H_2_O) and Europium (III) chloride hexahydrate (EuCl_3_·6H_2_O) were obtained from Sigma-Aldrich (St. Louis, MO, USA). A MON stock standard solution was prepared in acetonitrile at a concentration of 100 mg L^−1^; working solutions were obtained by dilution using water:methanol 15 + 85 *v/v*. All the solutions were stored at −20 °C when not in use.

### 3.2. LC-MS/MS Analysis for Moniliformin Determination

MON was extracted from cereal samples (10 g each) with 40 mL of a mixture acetonitrile:water 50 + 50 *v/v* using a rotary-shaking stirrer for 60 min. After filtration on a folded filter paper, the extract was diluted (1 + 1) with methanol:water 85 + 15 *v/v* and injected into the LC-MS/MS system (20 µL). The HPLC-MS/MS system consisted of a LC 1.4 Surveyor pump (Thermo Fisher Scientific, San Jose, CA, USA), a PAL 1.3.1 sampling system (CTC Analytics AG, Zwingen, Switzerland) and a Quantum Discovery Max triple quadrupole mass spectrometer; the system was controlled by an Excalibur 1.4 software (Thermo-Fisher). MON was chromatographed on a Supelcosil LC-NH_2_ column (75 × 3 mm, 3 µm, Supelco, Bellefonte, PA, USA) and separated using a gradient elution with 25 mM ammonium acetate containing 1.25 mM LaCl_3_·7H_2_O, and methanol as mobile phase A and B, respectively. The gradient program was linear gradient from 15% to 35% of solvent A in 3 min, then isocratic for 1 min; column conditioning lasted 7 min. The flow rate was 0.3 mL min^-1^. The ionisation was carried out with an ESI interface (Thermo-Fisher) in negative mode as follows: spray capillary voltage was 3.5 kV, sheath and auxiliary gas 40 and 15 psi, respectively; skimmer 9 V, temperature of the heated capillary 350 °C. The mass spectrometric analysis was performed in selected reaction monitoring (SRM). For fragmentation of the [M–H]^-^ ion (97 *m/z*), the argon collision pressure was set to 1.2 mTorr and the collision energy to 21 V. The detected and quantified fragment ion was 41 *m/z*. Quantitative determination was performed by an LC-Quan 2.0 software (Thermo-Fisher, Waltham, MA, USA).

### 3.3. HPLC-UV Analysis for Moniliformin Determination

After extraction as for LC-MS/MS analysis, a purification step was introduced before HPLC-UV analysis; a 2 mL aliquot of the extract was purified through a LC-NH_2_ column (500 mg, 3 mL, Supelco, Bellefonte, PA, USA), previously conditioned with 2 mL acetonitrile:water 50 + 50 *v/v*. The column was washed with acetonitrile (2 mL), deionized water (2 mL) and 1 mL of a 12.5 mM LaCl_3_·7H_2_O aqueous solution; then, MON was eluted in a graduated glass vial with additional 2 mL of the 12.5 mM LaCl_3_·7H_2_O aqueous solution. The purified extract was diluted (1 + 1) using acetonitrile and injected into a HPLC-UV system (30 µL). The HPLC system consisted of a Jasco PU 1580 pump (Jasco Corp., Tokyo, Japan) equipped with an AS 2055 sampling system and a UV 1575 detector set at 219 and 260 nm. The system was governed by a Borwin 1.5 software (Jasco). MON was chromatographed on a Supelcosil LC-NH_2_ column (250 × 3 mm, 5 µm, Supelco) and separated using a gradient elution with 2.5 mM LaCl_3_·7H_2_O (or TbCl_3_·6H_2_O) and acetonitrile as mobile phase A and B, respectively. The gradient program was 15% solvent A for 1 min, linear gradient to 35% solvent A in 3 min, then isocratic for 5 min; column conditioning lasted 7 min. The flow rate was 0.7 mL min^−1^; the column was thermostated at 25 °C.

### 3.4. Method Validation

For method validation, different parameters were considered. As regards LC-MS/MS, the matrix effect was examined; this effect is due to the presence of compounds that can co-elute, affecting the ionisation of the analyte. It was defined as the difference between the mass spectrometric response for the analyte in standard solution and the response for the same analyte at the same concentration in matrix extract. A comparison between the mass spectrometric response of MON in standard solution and in matrix extract at 100 µg l^−1^ was conducted; the matrix extract was evaluated by spiking an uncontaminated wheat or maize extract (950 μL) with MON standard (50 μL). Linearity of both HPLC-UV and LC-MS/MS measurement was established through five calibration standards in solvent, at concentrations between 2.5 and 250 µg L^−1^ for LC-MS/MS (20 and 2000 µg kg^−1^ for a cereal sample) and between 20 and 1000 µg L^−1^ (160 and 8000 µg kg^−1^ for a cereal sample) for HPLC-UV. The limit of detection (LOD) was defined as the level corresponding to a signal-to-noise ratio (S/N) of three, while the limit of quantification (LOQ) as the lowest level for which the repeatability of the analysis was below 10%. The decision limit (CCα) and the detection capability (CCβ) was calculated as reported by Commission Decision 2002/657/EC [21]. The accuracy of the proposed method was evaluated by determination of the recovery. Recovery experiments were performed by spiking wheat or maize flour in triplicate at two levels, 100 and 500 µg kg^−1^ for wheat and 250 and 1000 µg kg^−1^ for maize. The matrices were also analysed without spiking, as reagent blank. The spiked samples were allowed to stand for two hours at ambient temperature under a fume hood to allow any residual solvent to evaporate. Finally, the method repeatability was evaluated. Four samples (2 wheat and 2 maize flours) were extracted and analysed three times in different days.

### 3.5. Real Sample Collection and Analysis

A total of ten samples of maize and ten of wheat (2 durum wheat) were collected in northern Italy; samples were dried at 65 °C, milled using a cyclone hammer mill (1 mm sieve, Pulverisette, Fritsch GmbH, Idar-Oberstein, Germany) and homogenised. Then, an aliquot (1 kg) of the sample was taken and stored at −20 °C until the time of analysis. Quantification was carried out by LC-MS/MS, to detect lower contamination values.

## 4. Conclusions

Simple and suitable LC-MS/MS and LC-UV methods for MON determination were developed, fulfilling the wishes of the EFSA report. The simple addition of lanthanide ions to the mobile phase allowed an easy determination of MON using either mass spectrometric or UV detection. Data on MON occurrence in food and feed can be easily obtained using this method with a satisfactory accuracy. Further studies are needed to confirm the formation of mixed coordination complexes during the chromatographic separation. Finally, this chromatographic technique could be applied to the analysis of other mycotoxins, and also to the development of chiral separations.

## Figures and Tables

**Table 1:** Validation parameters of MON analysis using UV and LC-MS/MS detector (three replicates).

	HPLC-UV	LC-MS/MS
Matrix effect (at 800 µg kg^−1^) *	/	5% (wheat)
		8% (maize)
Calibration range *	160–8000 µg kg^−1^	20–2000 µg kg^−1^
LOD *	80 µg kg^−1^	10 µg kg^−1^
LOQ *	200 µg kg^−1^	25 µg kg^−1^
Average recovery		
Wheat	88.2% ± 5.5%	97.1% ± 4.3%
Maize	84.8% ± 5.4%	96.4% ± 5.1%

LOD: limit of detection; LOQ: limit of quantification. * Data referred to cereal sample.
